# Energyscapes and prey fields shape a North Atlantic seabird wintering hotspot under climate change

**DOI:** 10.1098/rsos.171883

**Published:** 2018-01-17

**Authors:** F. Amélineau, J. Fort, P. D. Mathewson, D. C. Speirs, N. Courbin, S. Perret, W. P. Porter, R. J. Wilson, D. Grémillet

**Affiliations:** 1 CEFE UMR 5175, CNRS – Université de Montpellier – Université Paul-Valéry Montpellier – EPHE, Montpellier, France; 2 Littoral Environnement et Sociétés (LIENSs), UMR 7266 CNRS-Université de La Rochelle, La Rochelle, France; 3 Department of Integrative Biology, University of Wisconsin, Madison, WI, USA; 4 Department of Mathematics and Statistics, University of Strathclyde, Livingstone Tower, 26 Richmond Street, Glasgow G1 1XQ, Scotland, UK; 5 Percy FitzPatrick Institute, DST/NRF Centre of Excellence, University of Cape Town, Rondebosch, South Africa

**Keywords:** bioenergetics, biologging, habitat modelling, little auk (*Alle alle*), migration, spatial ecology

## Abstract

There is an urgent need for a better understanding of animal migratory ecology under the influence of climate change. Most current analyses require long-term monitoring of populations on the move, and shorter-term approaches are needed. Here, we analysed the ecological drivers of seabird migration within the framework of the energyscape concept, which we defined as the variations in the energy requirements of an organism across geographical space as a function of environmental conditions. We compared the winter location of seabirds with their modelled energy requirements and prey fields throughout the North Atlantic. Across six winters, we tracked the migration of 94 little auks (*Alle alle*), a key sentinel Arctic species, between their East Greenland breeding site and wintering areas off Newfoundland. Winter energyscapes were modelled with Niche Mapper™, a mechanistic tool which takes into account local climate and bird ecophysiology. Subsequently, we used a resource selection function to explain seabird distributions through modelled energyscapes and winter surface distribution of one of their main prey, *Calanus finmarchicus*. Finally, future energyscapes were calculated according to IPCC climate change scenarios. We found that little auks targeted areas with high prey densities and moderately elevated energyscapes. Predicted energyscapes for 2050 and 2095 showed a decrease in winter energy requirements under the high emission scenario, which may be beneficial if prey availability is maintained. Overall, our study demonstrates the great potential of the energyscape concept for the study of animal spatial ecology, in particular in the context of global change.

## 1. Introduction

Theoretical and empirical studies have demonstrated that migration evolves to maximize fitness in a seasonal environment [1,2]. Migratory animals thereby target the most profitable areas [3], balancing their energy requirements with available resources, within ever-changing ecological landscapes. Such complex spatio-temporal match/mismatch of migratory species with their biotic and abiotic environments shape migratory dynamics and the fate of populations on the move [3,4]. Migrating animals are thus particularly vulnerable to climate change and resulting environmental modifications [3,5,6]. Among birds, most long-distance migrants breed at high latitudes, where climatic changes have the strongest amplitude and seasonally travel to more favourable wintering grounds [7,8]. Overall, this puts a strong emphasis on the migratory ecology of birds in a warming Arctic [9].

Bird sensitivity to climate change has been mainly studied during the breeding season. In particular, drastic changes in phenology have been noticed worldwide, triggered by shorter winters in polar and temperate regions [9–11]. Such phenological changes, that differ between species, can lead to a mismatch between food availability and demands for reproduction with strong impacts on breeding success, particularly for migrating species [5,10]. However, the impacts of climatic changes during winter have been the focus of fewer studies [6,12], probably because it is far more challenging to monitor individuals outside the breeding season [3].

A prerequisite to understanding how wintering animals are affected by climate change is to quantify the ecological benefits of seasonal habitat choice. For this purpose, it is essential to define and use ecological metrics that will allow researchers to rate and compare the profitability of wintering areas. According to evolutionary theory, these fitness proxies should show functional relationships with the capacity of each individual to survive and reproduce [13]. Indeed, a series of studies have identified the impact of wintering conditions on adult survival probabilities [14,15], while others have identified significant carry-over effects of such conditions on survival and reproduction in subsequent months [16], or even years [17].

Despite the great success and the necessity of such studies, they require long-term population monitoring and individual phenotyping, which are both extremely time-consuming and costly, particularly for long-lived species, like many migratory birds. Animal energetics offer a powerful, short-term alternative [18]. In particular, within the rapidly emerging field of movement ecology [19], the concept of energy landscapes (hereafter ‘energyscapes') seems extremely relevant and attractive for mechanistic explanations of animals' geographical distribution. Specifically, Wilson, Shepard and collaborators defined energyscapes as ‘environmentally dependent variation in the cost of transport, driven by variation in parameters such as incline, substrate type, vegetation, current speed, or direction' [20,21]. Here, we propose to broaden the concept of energyscape and define it as variation in the energy requirements of an organism across geographical space as a function of environmental conditions. In addition to the cost of transport, our definition thereby includes all costs associated with body maintenance and thermoregulation. Energyscapes are, consequently, highly sensitive to climatic conditions.

The recent development of mechanistic energetic models allows energyscapes to be estimated for any animal at any location around the globe [22–26]. This methodology, which in birds compares well with the accuracy of empirical measurements [27,28], provides a unique opportunity to develop and expand the concept of energyscapes for the study of animal migration.

In this study, we determined monthly energyscapes for an Arctic migrant, the little auk (*Alle alle*), and used them to test hypotheses related to wintering ecology in a climate change context. Little auks are the most numerous seabird of the Arctic (with an overall population estimated between 40 and 80 million individuals) and they are key components of Arctic marine food webs. Their winter migration across the North Atlantic has only recently been depicted through the use of miniaturized electronic geolocators [24]. Because the little auk is the smallest of all seabirds in the Atlantic Arctic, with a body mass of 150 g, its morphology makes it a particularly attractive model for the study of avian energetics in a migratory and wintering context. Indeed, its mass-specific resting metabolic rate is approximately seven times that of Emperor penguins (*Aptenodytes forsteri*) [29], and therefore energetic constraints acting upon little auk winter migratory decisions are predicted to be drastic. Finally, little auks feed on zooplankton, especially Calanoid copepods, which show a strong response to the climate-induced increase of North Atlantic surface water temperatures [30]. It is therefore an excellent model species for the study and forecast of the marine ecological consequences of rapid warming in the North Atlantic.

We took advantage of a large dataset on little auk migratory movements in the North Atlantic. This information was collected over 6 years using geolocators for a little auk population which breeds in East Greenland and predominantly overwinters off Newfoundland. Once the location of this wintering hotspot had been identified, we used the mechanistic model Niche Mapper™ and remote-sensing information to assess the energyscapes of birds within the North Atlantic. Then, we used resource selection functions (RSFs) to compare little auk spatial distribution with the modelled energyscapes and abundance of zooplankton prey. This approach allowed us to characterize the energetic strategy of wintering little auks, and to go further than the correlative approach of [24] in the definition of ecological drivers of their distribution. Finally, using climate forecasting models, we modelled the future energyscapes of little auks in their wintering region, to assess the potential impacts of forthcoming North Atlantic climate warming. As temperature is predicted to be the main driver of little auk energy requirements during winter according to [22], we expected that little auk energy demands would consequently decrease due to this warming.

We tested the hypotheses that (i) little auks optimize the position of their wintering location, so as to minimize their winter daily energy requirements, and maximize their use of zooplankton density gradients; and (ii) climate change will significantly modify little auk energy requirements at their current wintering location.

## 2. Methods

### 2.1. Seabird winter geolocation

Fieldwork took place at Ukaleqarteq (Kap Höegh, Liverpool Land, 70°44′ N, 21°35′ W), East Greenland. Breeding adults were equipped with light-level archival tags (GLS) each summer from 2009 to 2014 and recaptured the following years. All GLSs weighed between 0.8 and 1.5 g (0.6–1.1% of the lowest equipped bird weight). GLS types, technical characteristics and deployment/recapture details are available in electronic supplementary material, file S1. In total 244 GLSs were deployed, 102 were retrieved and 94 data files were exploitable. Three birds were equipped for 2 years, and one bird for 3 years. Birds were captured either in their underground nests or with a lasso placed on the rocks surrounding their nests. They were weighed and fitted with a metal ring on which a GLS was attached using a cable tie. Previous investigations showed that GLS deployments had no measurable impacts on little auk body condition [24].

GLS data were analysed with British Antarctic Survey (BAS) softwares (TransEdit and Locator) for BAS and Biotrack devices, and with Intiproc^®^ for Migrate Technology devices. In both cases, the light intensity threshold was set to 10 lux, and the sun elevation angle to −3°. Two positions per day were obtained for each bird, one at local noon and one at local midnight. Points on land and outside the study area (70° W–15° E, 30° N–80° N) were removed. All bird handling procedures were approved by the Government of Greenland (Permits nos. 66.01.13, 2011–047447, 2012-065815, 2013-083634 and 2014-098814) and validated by the ethics committee of the French Polar Institute (Permit no. MP/53/06/12). Tracking data are available on Movebank (https://www.movebank.org/) under the study named ‘Adaclim'.

### 2.2. Modelling seabird energyscapes

To model seabird energy requirements at a point in time and space, we used the mechanistic model Niche Mapper™, which evaluates the daily energy requirements of an individual using the biophysical properties of seabird bodies exposed to specific microclimatic conditions [22,31]. Niche Mapper consists of two submodels: a microclimate model and an animal model. The microclimate model uses macroclimate data (sea surface temperature (SST), air temperature, cloud cover, relative humidity and wind speed), substrate properties, geographic location and time of year to calculate hourly environmental conditions at the animal's height. Microclimate model calculations are detailed in [32]. The animal model uses the outputs from the microclimate model to iteratively solve a heat balance equation to find the metabolic rate needed for the animal to maintain its body temperature, accounting for convective, radiative, evaporative and solar heat fluxes with its microenvironment. Heat balance calculations are detailed in [33]. Little auks were modelled as a series of simple shapes that have well-understood heat transfer properties that enable surface temperature calculations—and thus heat flux calculations—given a certain core temperature to maintain: two ellipsoids for the head and torso and two cylinders for the featherless parts of the legs. Legs were modelled to allow heat loss, with possible changes in peripheral blood flow as a function of bird heat balance and external temperature. For time not spent diving or flying, little auks were modelled as floating on the ocean surface with legs and 25% of torso submerged in the water (electronic supplementary material, file S2). Auks were modelled as diving 24% of each day and flying 9% of each day (electronic supplementary material, file S2, [22]). All model input values are detailed in electronic supplementary material, file S2. Monthly average environmental input data were downloaded from the International Comprehensive Ocean-Atmosphere Data Set (ICOADS, http://icoads.noaa.gov/, observed data, 1° × 1° resolution). The model was run to predict little auk daily energy requirements during the winter months (November, December, January and February) between 30° N to 80° N and 70° W to 15° E with a 1° × 1° grid size. A sensitivity analysis was performed to identify input variables which had the strongest influence upon modelled energy requirements (electronic supplementary material, file S3).

### 2.3. Zooplankton winter abundance

We used the distribution and abundance of the copepod *Calanus finmarchicus*, one of little auk main prey, as a proxy for little auk prey availability [24,34]. Since temporally continuous observational data on *C. finmarchicus* are not available on the spatial scale required for our study, we used abundance estimates obtained from the ocean-scale population model of [35]. This is an explicitly spatial model (at 0.5° × 0.25° grid size) where at each discrete location the population is divided into surface (0–100 m depth) individuals and deep diapausing individuals. The surface population is further divided into discrete development classes that map on to the naupliar and copepodite stages. Development rate and egg production depend on the local temperature and food availability. A proportion of individuals entering the pre-adult (CV) copepodite stage join the diapausing population and emerge as surface adults (CVI) in the spring. Temperature and spatial transport for the model was determined using outputs from the Ocean Circulation and Climate Advanced Modelling Project [36] ocean circulation model, while phytoplankton food was estimated from SeaWIFS satellite observations (https://oceancolor.gsfc.nasa.gov/data/seawifs/). Model outputs have been successfully compared with field data from the continuous plankton recorder, *in situ* winter distributions of diapausers and, at some locations, copepod time series. A full formal description of the approach is given in [35]. The spatial extent of the model encompasses our entire study area, as defined in the previous section, and we used the modelled abundances of the surface CV and CVI copepodite stages as our proxy for little auk prey. The current version of this model does not yet allow forward projections of *C. finmarchicus* abundance due to a lack of reliable projections of phytoplankton abundances.

### 2.4. Habitat selection of little auk

**Figure 1.**
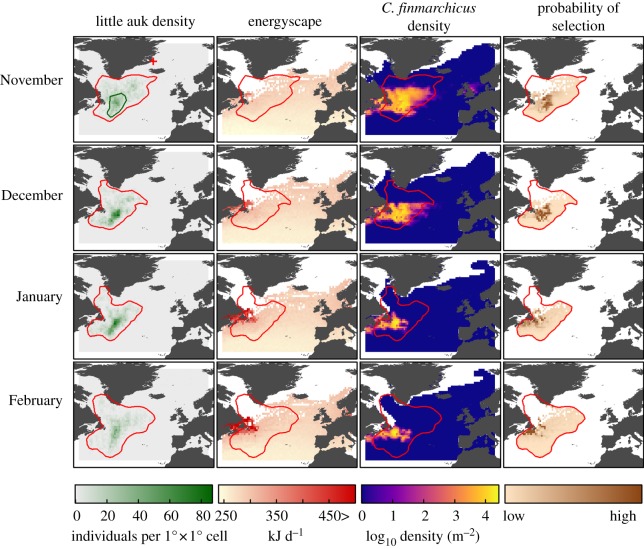
Little auk winter distribution (number of individuals per 1° × 1° cell, green), little auk energyscape (kJ d^−1^, red), little auk prey distribution (log _10_ density (m^−2^)) and relative probability of habitat selection by little auks for each month in the North Atlantic (brown). Monthly 95% kernel contours of little auk positions are presented in red. The position of the colony (red plus symbol) and the 50% kernel of winter positions (green) are presented in the top-left panel. White areas represent an absence of data.

We determined how little auks balanced the cost of their daily energy requirements with the benefits of searching for *C. finmarchicus* for each winter month (November to February). We used RSFs [37] that assessed whether a given habitat feature is used disproportionately relative to availability (i.e. selection or avoidance). RSFs compared the daily energy requirements of little auks and the density of *C. finmarchicus* at GLS locations with those expected at an equal set of random locations generated within the 95% kernel density of observed locations for each month (figure 1). This kernel density contour was chosen to represent available locations (both favourable and less favourable wintering locations). RSFs were fitted using generalized linear mixed models with a binomial distribution for errors and a logit link. We added a random intercept to account for the unbalanced number of locations collected across individuals [38]. We assessed empirical standard errors that are robust to both among- and within-individual correlations (i.e. serial correlation) and that provide robust estimates of significance [39]. The RSF took the form $w({\text{x}}_{ij})=\exp(\beta_{0}+\beta_{1}x_{1ij}+\beta_{2}x_{2ij}+\cdots +\beta_{n}x_{nij}+\gamma_{0j})$, where $w({\text{x}}_{ij})$ is the relative probability of selection for little auk, **x** is a vector, *β_0_* is the mean intercept, *β* is the estimated fixed regression coefficient for continuous covariate *x*, *i* represents the *i*th observation, *j* represents the *j*th individual and *γ*_0*j*_ is the random effect on the intercept *β_0_* for animal *j*. We included the squared energy requirements and the squared density of *C. finmarchicus* to allow for quadratic effects, and an interaction term between prey density and energy requirements. Both the daily energy requirements and density of *C. finmarchicus* were centred to avoid collinearity issue and multicollinearity was low in all RSFs (variance inflation factors ≤6.6 in all models, [40]). We evaluated model robustness using k-fold cross-validation, by developing RSFs with 80% of the locations (training set), and then by testing the predictive power of these RSFs with the 20% withheld locations (testing set) [39,41]. RSFs were performed with the GLIMMIX procedure of SAS v. 9.2 software (SAS Inst.).

### 2.5. Energyscape projections

We investigated changes in energy requirements within the core wintering area of little auks (defined as the 50% kernel density contour of the positions from 1 November to 28 February) for three decades centred in 2010, 2050 and 2095, using climatic projections based on two IPCC (Intergovernmental Panel on Climate Change) scenarios: one reflecting a low greenhouse gas concentration trajectory (Representative Concentration Pathway, RCP 2.6) and one reflecting a high concentration trajectory (RCP 8.5). Based on model comparisons by [42] for our studied area, we chose the Canadian Earth System Model v. 2 (CanESM2), which reproduced the best observed climatic trends off Newfoundland. Climatic predictions for sea-surface temperatures, air temperatures, cloud cover and relative humidity were downloaded from http://www.cccma.ec.gc.ca/data/cgcm4/index.shtml. As Niche Mapper™ requires a minimum and a maximum value for each input variable, and only mean values were available from CanESM2, we calculated min/max from the observed amplitude within the same variables from ICOADS data. Niche Mapper™ was run for each year of the three decades using both scenarios (RCP 2.6 and 8.5). Then, for each month and each run, we calculated the mean daily energy requirements of little auks within their core wintering area (figure 4, core wintering area presented in the top-left panel of figure 1, [43]).

## 3. Results

### 3.1. Little auk winter distribution and timing of migration

**Figure 2.**
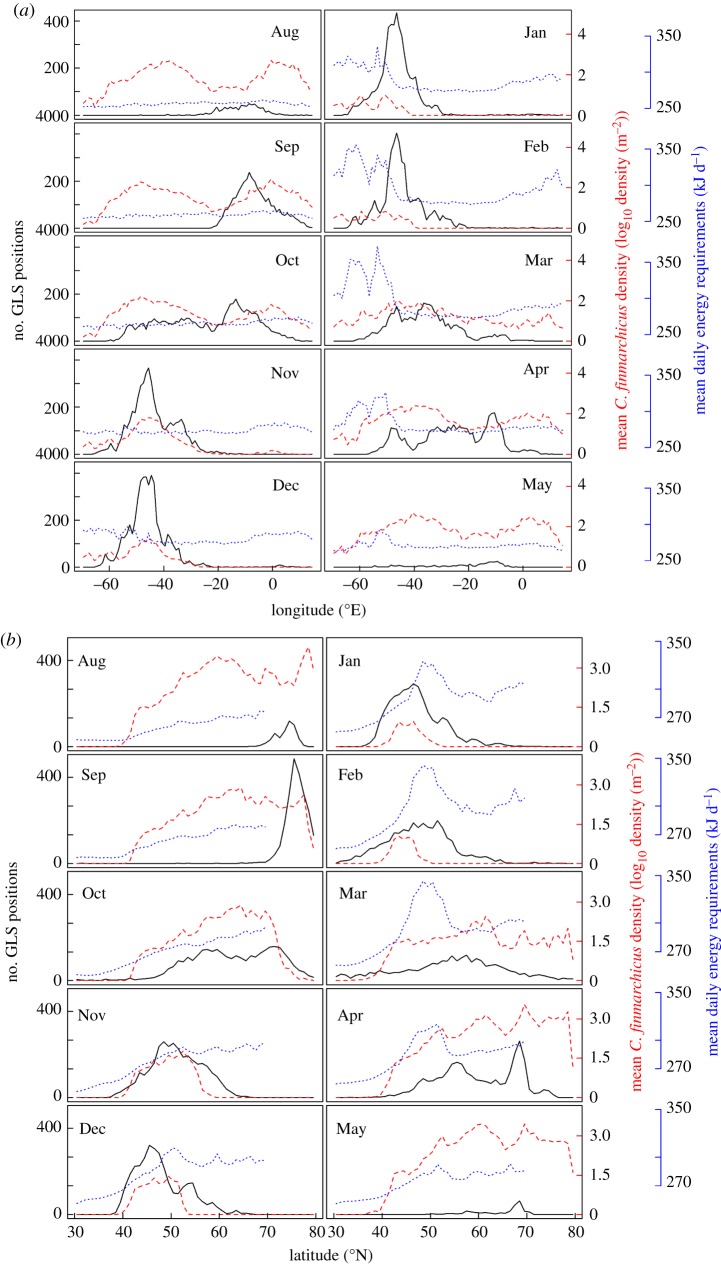
Number of little auk GLS positions (black), mean *Calanus finmarchicus* density in log_10_ density m^−2^ (red, dashed line) and little auk mean daily energy requirements in kJ d^−1^ (blue, dotted line) per longitude (*a*) and latitude (*b*) and per month. The numbers of positions are low in August and May because most of the birds are already above the polar circle and permanent daylight does not allow positioning using GLS recorders. Similarly, bird position is not indicated during the summer (June and July), but their position was known as they were breeding at our study site in East Greenland.

Figure 1 presents the density of bird positions per 1° × 1° cell for each winter month (wintering period). On figure 2, bird positions were summed by longitude and latitude for each non-breeding month (migration + wintering periods). Over the study period (2009–2015), GLS recordings showed that little auks migrated to their wintering grounds in October (figure 2). Birds wintered off Newfoundland, between 40° N and 55° N and 35° W and 55° W, at nearly 3000 km from their colony. They remained within this area until February, and migrated towards their breeding grounds in March (figure 2). Figure 2 shows a clear overlap between little auk distribution and peaks of *C. finmarchicus* abundance over the winter months. Hence, the timing of migration matched the narrowing (October) and broadening (March) of the *C. finmarchicus* distribution in the North Atlantic.

### 3.2. Little auk energyscape

The sensitivity analysis revealed that little auk daily energy requirements are mainly driven by SSTs and air temperatures (electronic supplementary material, file S3). Little auk daily energy requirements in the North Atlantic followed a latitudinal gradient, with higher energy requirements at higher latitudes all year round (figures 1 and 2*b*). During winter, daily energy requirements were highest around Newfoundland, within the cold Labrador Current, and increased gradually from December to March. Visually, this peak of seabird energyscape overlapped with the highest predicted densities of *C. finmarchicus*, especially in January and February, where the prey spatial distributions were less widespread (figure 1). Consequently, in January and February, the little auk distribution became slightly uncoupled from the prey distributions, birds remaining in areas with lower energy requirements in the southeast of prey distribution (figures 1 and 2*b*).

### 3.3. Little auk habitat selection

**Figure 3.**
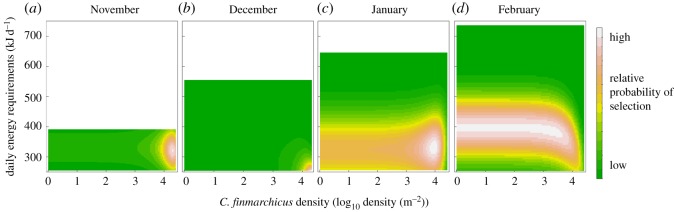
Relative probability of selection for little auk as a function of their daily energy requirements, and *C. finmarchicus* density modelled for each during the wintering period. Little auk daily energy requirements are only represented in the range of observed values for each month.

All RSFs were robust to k-fold cross validation and had a high power to predict spatial distribution of little auk (table 1). From November to January, little auks had a higher likelihood of selecting areas with high *C. finmarchicus* densities and moderate levels of daily energy requirements (figure 3*a–c*). In December, little auks were more likely to occur where their energy requirements were around 260 kJ d^−1^. In November and January, they experienced increased daily energy requirements, around 340 kJ d^−1^. As the daily energy requirements progressively increased, and the prey spatial distribution strongly overlapped high energyscapes around Newfoundland in January and February (figures 1 and 2), little auk made a trade-off between the benefit of food resource and energy requirements. In January, little auks also selected in a lesser extent areas with low density of *C. finmarchicus* (figure 3*c*). The trade-off was still stronger in February, little auks being more likely to select areas with high daily energy requirements (figures 1–3*d*).

**Table 1:** Generalized linear mixed effect models for habitat selection for little auk (*n *= 94), with parameter estimates (*β*) and their 95% confidence interval (CI) for each winter month. *Calanus finmarchicus* density (Cfin, log_10_ density m^−2^) and energy requirements (ER) were centred. 95% CI exclude 0 at *α* <0.05 (*), <0.01 (**), <0.001 (***).

	November *β* 95% CI	December *β* 95% CI	January *β* 95% CI	February *β* 95% CI
intercept	0.073 ± 0.088	0.31 ± 0.077***	0.32 ± 0.11**	0.36 ± 0.11**
Cfin	0.19 ± 0.031***	0.20 ± 0.030***	0.081 ± 0.029**	0.10 ± 0.046*
Cfin^2^	−5.8 × 10^−3 ^± 1.8 × 10^−3^**	−7.7 × 10^−3^ ± 2.1 × 10^−3^***	−5.3 × 10^−3 ^± 1.4 × 10^−3^***	−5.7 × 10^−3 ^± 2.3 × 10^−3^*
ER	0.017 ± 7.5 × 10^−3^*	−4.3 × 10^−3 ^± 6.1 × 10^−3^	3.0 × 10^−3 ^± 4.2 × 10^−3^	0.016 ± 3.9 × 10^−3^***
Er^2^	−2.0 × 10^−3 ^± 8.7 × 10^−5^*	−4.0 × 10^−5 ^± 4.1 × 10^−5^	−7.0 × 10^−5 ^± 4.3×10^−5^	−9.0 × 10^−5 ^± 3.3 × 10^−5^**
Cfin * ER	−2.0 × 10^−4 ^± 1.4 × 10^−3^	−1.8 × 10^−3 ^± 6.4 × 10^−4^**	−1.1 × 10^−4 ^± 2.6 × 10^−4^	−1.0 × 10^−3 ^± 2.7 × 10^−4^***
k-fold (*r*_s_)	0.93 ± 0.039	0.87 ± 0.058	0.90 ± 0.046	0.91 ± 0.041

### 3.4. Predictions

**Figure 4.**
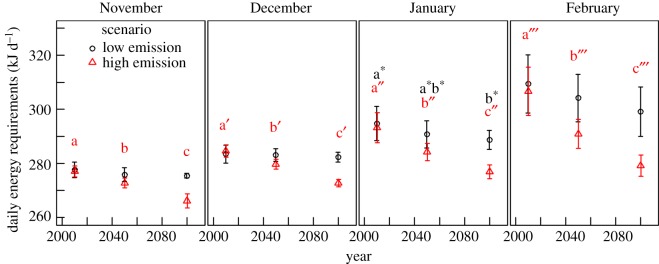
10-year average little auk energetic requirements in 2010, 2050 and 2095 for two emission scenarios. Values are means ± s.d. Energetic requirements are calculated for little auk core wintering areas, defined as the 50% kernel density contour of the GLS positions obtained each year (2009–2015) from 1 November to 28 February (represented in top-left panel of figure 1). Low emission = RCP 2.6, black, circles. High emission = RCP 8.5, red, triangles. For each scenario and month, means with different letters are significantly different (Tukey post hoc test, table 2). No difference was found for RCP 2.6 in November, December and February (table 2).

Under the low emission scenario, projections of little auk energy requirements did not differ with time, except in January where a slight difference between 2010 and 2095 was found (figure 4, table 2, November: *F*_2,27 _= 2.69, *p *= 0.086, December: *F*_2,27 _= 0.49, *p *= 0.61, January: *F*_2,27 _= 3.7, *p *= 0.038, February: *F*_2,27 _= 2.88, *p *= 0.074). Under the high emission scenario, there was a significant decrease in energy requirements with time, for each month (figure 4, table 2, November: *F*_2,27 _= 63.5, December: *F*_2,27 _= 99.1, January: *F*_2,27 _= 42.8, February: *F*_2,27 _= 46.3, all *p *< 0.0001).

**Table 2.** One-way ANOVAs and Tukey post hoc tests comparing mean energy requirements per decade, for each scenario and each month. d.f., degrees of freedom; SS, sum of squares; MS, mean square.

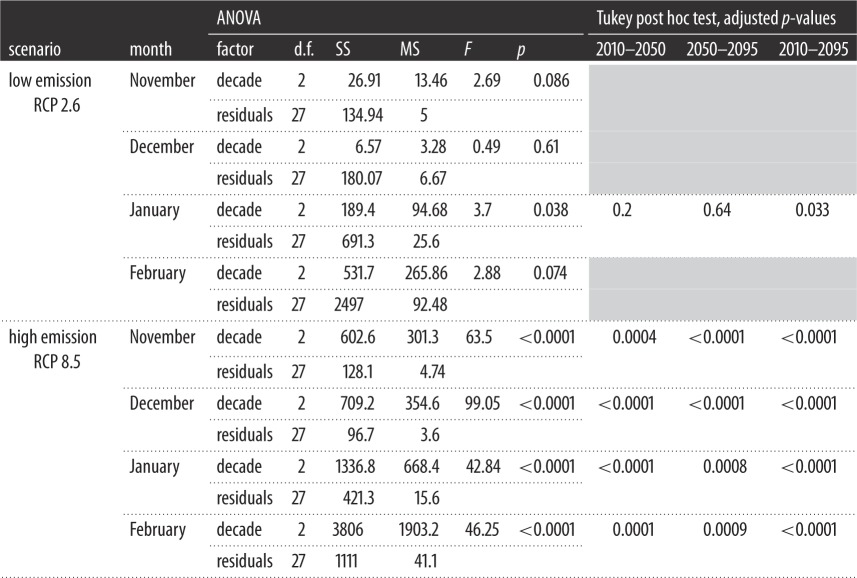

## 4. Discussion

Our detailed analysis of little auk wintering energyscapes and prey fields in a climate change context, support our first hypothesis: the wintering grounds of little auks were shaped by both prey availability and energyscapes. Yet, prey fields seemed more important than energy requirements to explain bird winter locations. Our results also partially support our second hypothesis: forthcoming climate warming will substantially modify energyscapes for little auks wintering off Newfoundland under a high emission scenario only. Overall, our study highlights the relevance of the emerging energyscape concept as a methodological framework for the study of animal migration ecology and evolution in the context of global change [44]. Nevertheless, our results also demonstrate the need to include prey fields and their energetic profitability in future energyscape studies.

### 4.1. Seabird wintering in the Northwest Atlantic

In this study, we took advantage of existing, detailed knowledge of Calanoid copepod distribution and habitat models in the North Atlantic [35,45]. While the importance of *Calanus finmarchicus* in the winter diet of little auks is still a matter of debate, we considered that *C. finmarchicus* density and distribution was a good proxy for overall little auk prey availability, as they fed either on those prey, or on species from the next trophic level, such as amphipods and krill when they wintered off Newfoundland [31,46,47]. Interestingly, little auk timing of migration correlated closely with *C. finmarchicus* range restriction in autumn, and range expansion in spring (figure 2). This observation also strongly suggests a tight link between little auks and *C. finmarchicus* off Newfoundland in winter.

Little auks did not winter in areas where their energyscape was the most favourable, i.e. the East Atlantic (figure 1). In fact, they were constrained because highly productive areas were also located where their energy requirements are higher, within and around the cold Labrador Current off Newfoundland (figures 1 and 2). Within this area, they fine-tuned their spatial distribution in respect to the energyscape, targeting the southeastern range of *C. finmarchicus* during winter, where prey items were still abundant and bird energyscapes were more advantageous (figures 1 and 2). They seemed to avoid shelf waters, potentially because at these locations cold water stemming from the Labrador Current [48] increased their energy requirements. As in summer, they also targeted the edges of the continental shelf such as around the Grand Banks, where upwelling concentrates prey [49]. A second wintering strategy was also observed: according to the RSF, there was a medium (January) and high (February) probability of little auk occurrence with medium energy requirements when there was little or no *C. finmarchicus* (figure 3*c,d*), which suggests that some of the little auks from East Greenland could rely on different prey/food web at the end of winter.

Little auk wintering areas are also crucial for many other seabird species [50–57]. Indeed, the Northwest Atlantic is a major wintering hotspot for seabirds from various breeding grounds [50–53], and even for birds from the Southern Hemisphere during the boreal summer [57]. It is also a migration stopover for some long-distance migrants [55,56], and Southwest Greenland itself hosts seabirds from the whole Arctic [54]. Therefore, the energyscape concept illustrated here, as well as associated analytical tools, will also be of great use for the general ecological understanding of the evolution of aquatic bird migration in the North Atlantic, in a global change context.

### 4.2. Wintering strategies under forecasted climate change

Under the high emission scenario (RCP 8.5), little auk energy requirements should decrease during the twenty-first century within their current core wintering areas (figure 4, table 2). Under the low emission scenario (RCP 2.6), their energy requirements are predicted to decrease only in January (table 2, figure 4). The RCP 2.6 represents a high mitigation scenario that aims to keep global warming to less than 2°C above pre-industrial temperatures [7]. A decrease in energy requirements during winter is therefore likely for little auks, and could be beneficial for them as well as for other wintering seabirds. In order to anticipate the future distributions of wintering migrants, it is also necessary to know how their prey will react to ongoing environmental changes, and two scenarios can be considered: (1) if prey biomass remains constant, winter visitors could stay in their current wintering areas, especially if those become energetically more profitable, due to climate warming. However, (2) if prey abundance decreases in this area, they may have to move to follow their current prey distribution and/or target different prey species. It is therefore imperative to understand whether little auk wintering areas off Newfoundland will remain highly productive during winter, thereby supporting scenario (1), or if changes in ocean circulation may modify primary productivity and associated food webs. Higher temperatures are known to favour smaller zooplankton species [58,59]. Therefore, prey biomass has to be maintained to sustain the rest of the food web, including migratory bird populations. Yet, over the past 40 years, a decrease in total zooplankton biomass has been observed in the Northeast Atlantic and the same phenomenon is predicted to occur in the Northwest Atlantic [60].

Thanks to the continuous plankton recorder survey [61], the spatio-temporal evolution of zooplankton species has been well studied in the North Atlantic during the last 50 years. A northward shift in zooplankton assemblages and in the copepod *Calanus finmarchicus* has been demonstrated for the Northeast Atlantic [45,62] as well as for the Northwest Atlantic [63], whereas no changes have been found so far for the Arctic species *C. glacialis* and *C. hyperboreus* that form an important food link at higher latitudes [62]. The authors speculate that this may be due to the southward penetration of water from the Labrador current into the Northwest Atlantic [62]. These studies suggest that seabird visitors may have to move northwards to target their preferred prey during winter. It is, however, not clear whether prey will move further north in the Labrador Sea. Recent climatic models suggest a cooling of the Labrador Sea due to a local collapse of deep-ocean convection [64] that would prevent *Calanus finmarchicus* range expansion.

Currently, seabirds (including little auks) that winter at higher latitudes in the Labrador Sea may rely on different prey communities [31,47,65,66]. Indeed, energy requirements are higher further north [22], but prey of these areas are richer in lipids [67,68]. In particular, copepods from colder waters are bigger and richer in lipids [63]. Changes in winter distribution will be facilitated in species that show a low individual consistency in their wintering areas. While seabirds were until recently seen as highly consistent in their wintering locations [69], this understanding is changing [70], and among Alcids in particular, individual consistency varies strongly between species, suggesting some plasticity in wintering ecology [71,72]. In this study, preliminary results on little auks equipped with GLSs during two or three successive years confirm that they are not always wintering at the same place (electronic supplementary material, file S4).

Finally, a northward shift of little auk wintering grounds could be beneficial because it reduces overall migration distance. In the case of little auks from East Greenland, wintering in the Labrador Sea instead of the area around the Grand Banks of Newfoundland would reduce migration distance by about 1000 km, corresponding to 33% of their current migration distance. Such a shortening in migration distance has been observed in some terrestrial bird species [73,74], and an experimental study has even shown that residency can evolve rapidly in a passerine population if selection pressure towards shorter migration is maintained [75]. Migrating closer to the breeding area is not only beneficial because of a decrease in travel costs, but also because it allows a better detection of the environmental conditions occurring at the breeding grounds, so as to match resource phenology at the breeding site [74].

However, one potential barrier to the poleward shift of little auk wintering areas is the decrease in daylight duration and the polar night, but this does not seem to directly impact Arctic seabirds, as individual seabirds, including little auks, have been found to actively forage during the polar night in Greenland, Spitsbergen or the Barents Sea ([76,77], Fort J *et al*. unpublished). Yet the polar night also limits winter primary productivity at high latitudes, as well as the carrying capacity of the coastal ecosystem with respect to apex predators. In this context, poleward range expansions may also enhance inter- and intra-specific competition [78,79]. In our study system, little auks from other populations are already wintering further north in the Labrador Sea, and could become competitors if the whole wintering area of this species is narrowed [80]. Lastly, a poleward shift may result in a mismatch between prey blooms and the timing of migration, as phytoplankton blooms occur later at higher latitudes because of light restriction during winter [81].

### 4.3. Outlook

With the present study, we have broadened the concept of energy landscapes as defined by Wilson, Shepard and collaborators [20,21], and redefined it as the spatial variations in energy requirements of an animal at a specific moment in time. Therefore, in contrast to [21], this approach does not require recording detailed accelerometry data to estimate transport cost for each study individual, but rather some knowledge of environmental conditions encountered through time, and on species time-budgets and metabolism. Energyscapes calculated with Niche Mapper™ can consequently be used in a much wider range of species and ecological contexts [82,83]. These two approaches could nonetheless be used complementarily if accelerometry data help fine-tune NicheMapper™ input parameters.

Our study of little auk wintering ecology has also demonstrated the overarching importance of prey fields, for a thorough understanding of individual strategies and population biogeography under climate change. Therefore, once functional relationships between prey availability and predatory performance become known for little auks [84], we propose further expanding the energyscape concept to include this information. Energyscapes would then be defined as the energetic profitability, for a given species at a given time, thereby setting an exciting target for future investigations of species biogeography in a changing world.

## Supplementary Material

Details of GLS deployments

## Supplementary Material

Summary of parameters used in Niche Mapper

## Supplementary Material

Results of the sensitivity analysis in Niche Mapper for the daily energy requirements of little auks in winter

## Supplementary Material

Winter locations of four birds for which 2 (or 3) consecutive years were recorded.

## Data accessibility

Our data are deposited on Movebank (https://www.movebank.org/) under the study named ‘Adaclim'.
