# Supplementary material for: Energyscapes and prey fields shape a North Atlantic seabird wintering hotspot under climate change
Source: R Soc Open Sci. 2018 Jan 17;5(1):171883. doi: 10.1098/rsos.171883 (PMC5792952; doi:10.1098/rsos.171883)
Supplement: Details of GLS deployments [file rsos171883supp1.pdf]

Energyscapes and prey fields shape a North Atlantic seabird wintering hotspot under climate change. Amélineau F., Fort J., Mathewson P.D., Speirs D.C., Courbin N., Perret S., Porter W.P., Wilson R.J., Grémillet D. **Royal Society Open Science**.

ESM file 1: Details of GLS deployments.

**Year**	**GLS type**	**Manufacturer**	**Dimensions**	**Weight (g)**	**Deployed**	**Retrieved (year+1)**	**Included in the analyses**
2009	Mk14	BAS	20x8.5x5.5	1.5	88	51	47
2010	Mk18L	BAS	15x10x6	1.5	16	7	7
2010	Mk12	BAS	20x8x4	0.8	16	12	8
2011	Mk10b	BAS	17x9x6	1.1	30	12	12
2012	Mk4093	Biotrack	15x10x6	1.5	30	8	8
2013	Mk4093	Biotrack	15x10x6	1.5	30	4	4
2014	Mk4093	Biotrack	15x10x6	1.5	19	2	2
2014	Intigeo C65	Migrate technology	14x8x6	1.0	15	6	6
				**total**	**244**	**102**	**94**
