# Supplementary material for: Energyscapes and prey fields shape a North Atlantic seabird wintering hotspot under climate change
Source: R Soc Open Sci. 2018 Jan 17;5(1):171883. doi: 10.1098/rsos.171883 (PMC5792952; doi:10.1098/rsos.171883)
Supplement: Summary of parameters used in Niche Mapper [file rsos171883supp2.pdf]

Energyscapes and prey fields shape a North Atlantic seabird wintering hotspot under climate change. Amélineau F., Fort J., Mathewson P.D., Speirs D.C., Courbin N., Perret S., Porter W.P., Wilson R.J., Grémillet D. **Royal Society Open Science**.

ESM file 2: Parameters used in Niche Mapper™

**Morphological properties**	Value	References
Body mass (g)	152	This study
Body plumage depth (dorsal-ventral) (mm)	7.4–12.8	Fort et al 2009
Head plumage depth (d-v) (mm)	5.2-9.4	This study
Plumage reflectivity (d-v) (%)	40.4–65.0	Fort et al 2009
Body feather length (d-v) (mm)	20.0–19.2	Fort et al 2009
Head (feather length (d-v) (mm)	11.6-10.7	This study
Feather diameter (d-v) (µm)	33.0–33.0	Fort et al 2009

**Physiological properties**	Value	References
Body core temperature (°C)	40	Gabrielsen et al 1991
Flesh thermal conductivity ($W.m^{-1}.°C^{-1}$)	0.5-2.8	Cheng and Plewes 1992
Oxygen extraction efficiency (%)	35	Fort et al 2009
Bird density ($kg.m^{-3}$)	932.9	Fort et al 2009
Resting metabolic rate (W)	2.02	Gabrielsen et al 1991
Flight metabolism (W)	10.57	Fort et al 2009

**Behavioural properties**	Value	References
Ventral area contacting substrate (%)	25	Fort et al 2009
Proportion of time spent flying per day (%)	9	Fort et al 2009, 2010
Proportion of time spent diving per day (%)	24	Fort et al 2010

**Environmental data**	source
Sea surface temperature (°C)	ICOADS (http://icoads.noaa.gov/)
Air temperature (°C)	ICOADS (http://icoads.noaa.gov/)
Cloud cover (%)	ICOADS (http://icoads.noaa.gov/)
Relative humidity (%)	ICOADS (http://icoads.noaa.gov/)
Wind speed ($m.s^{-1}$)	ICOADS (http://icoads.noaa.gov/)
