# Supplementary material for: Energyscapes and prey fields shape a North Atlantic seabird wintering hotspot under climate change
Source: R Soc Open Sci. 2018 Jan 17;5(1):171883. doi: 10.1098/rsos.171883 (PMC5792952; doi:10.1098/rsos.171883)
Supplement: Results of the sensitivity analysis in Niche Mapper for the daily energy requirements of little auks in winter [file rsos171883supp3.pdf]

Energyscapes and prey fields shape a North Atlantic seabird wintering hotspot under climate change. Amélineau F., Fort J., Mathewson P.D., Speirs D.C., Courbin N., Perret S., Porter W.P., Wilson R.J., Grémillet D. **Royal Society Open Science**.

ESM file 3: Results of the sensitivity analysis in Niche Mapper™ for the daily energy requirements of little auks in November, December, January and February.

Varname	Range	Variation of energy requirements (%)
body T	±10%	-2.56
plumage reflectivity	±10%	0
feather length	±10%	-1.63
feather diameter	±10%	0.61
plumage density	±10%	-0.98
plumage depth		
activity E released at heat	±10%	0.5
flight metabolism	±10%	0
ventral area on water	±10%	0
cloud cover	min-max	-0.45
relative humidity	min-max	-0.16
SST	min-max	-11.4
Air temperature	min-max	-8.54
wind	min-max	0.89
