# Supplementary material for: Energyscapes and prey fields shape a North Atlantic seabird wintering hotspot under climate change
Source: R Soc Open Sci. 2018 Jan 17;5(1):171883. doi: 10.1098/rsos.171883 (PMC5792952; doi:10.1098/rsos.171883)

Energyscapes and prey fields shape a North Atlantic seabird wintering hotspot under climate change. Amélineau F., Fort J., Mathewson P.D., Speirs D.C., Courbin N., Perret S., Porter W.P., Wilson R.J., Grémillet D. **Royal Society Open Science**.

ESM file 4: Winter locations of four birds for which 2 (or 3) consecutive years were recorded. For each winter (November, December, January and February) the 50% kernel of each bird is presented: (a) Bird 3645. (b) Bird 3656. (c) Bird 3668. (d) Bird 3679.

(a)

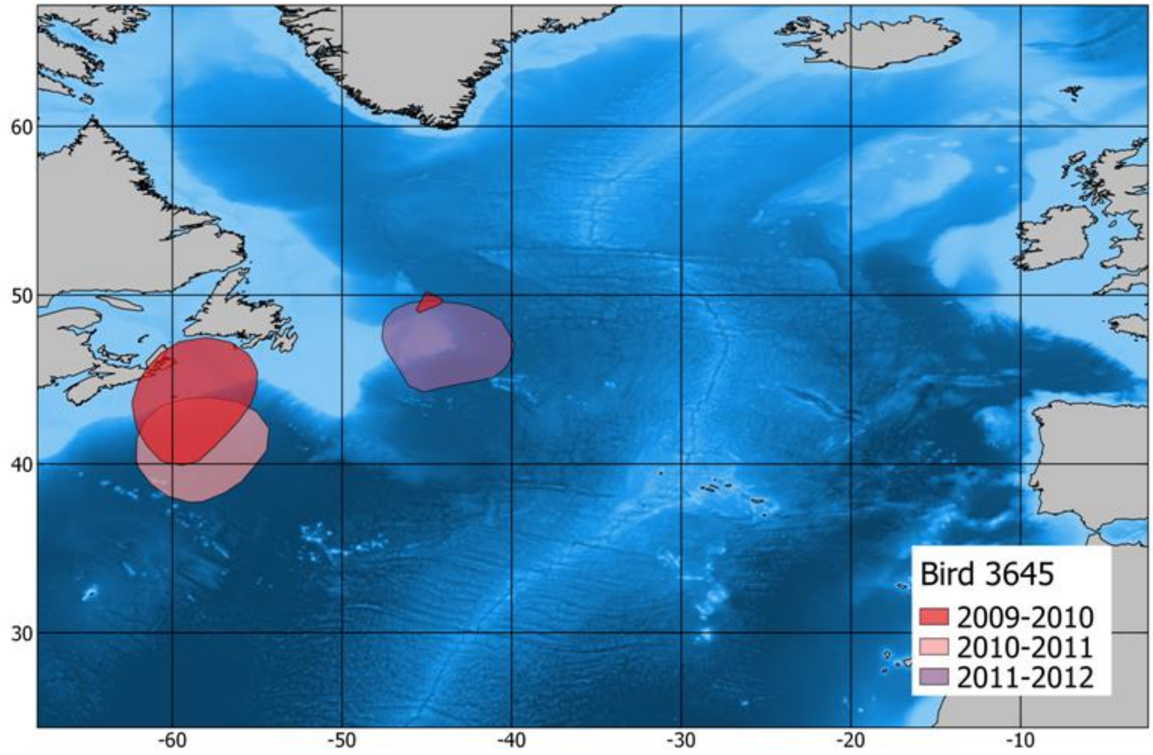

(b)

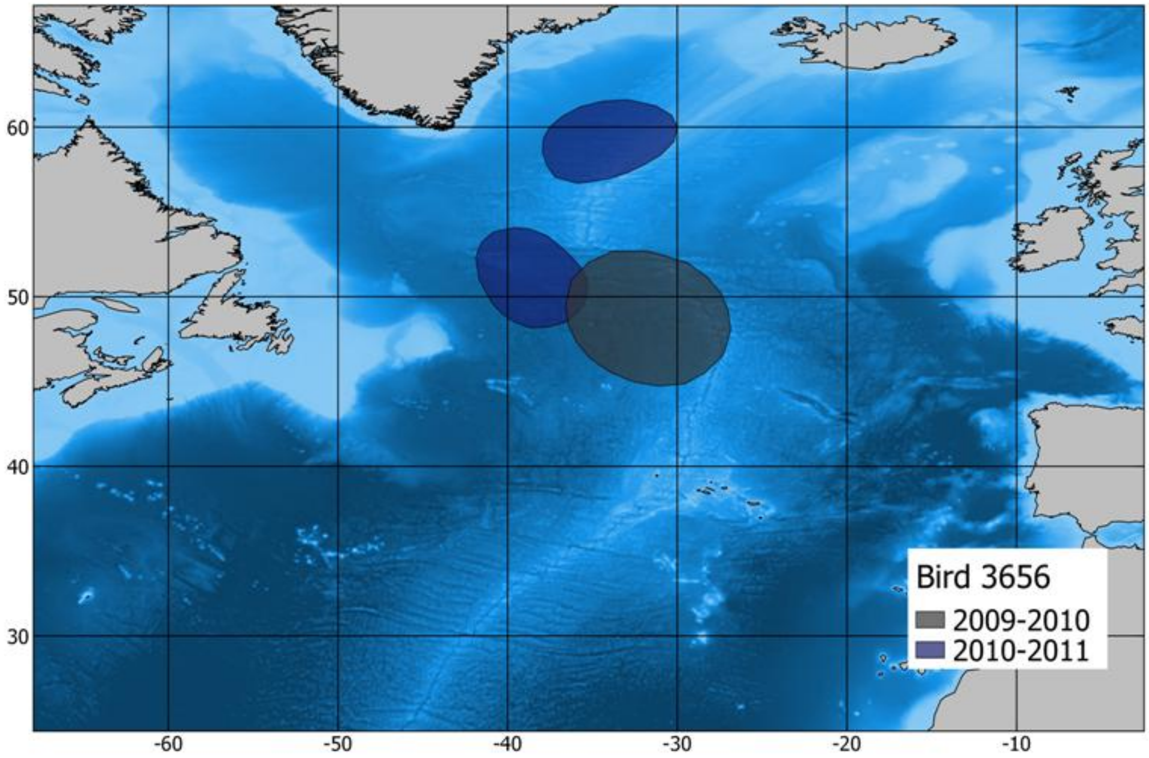

(c)

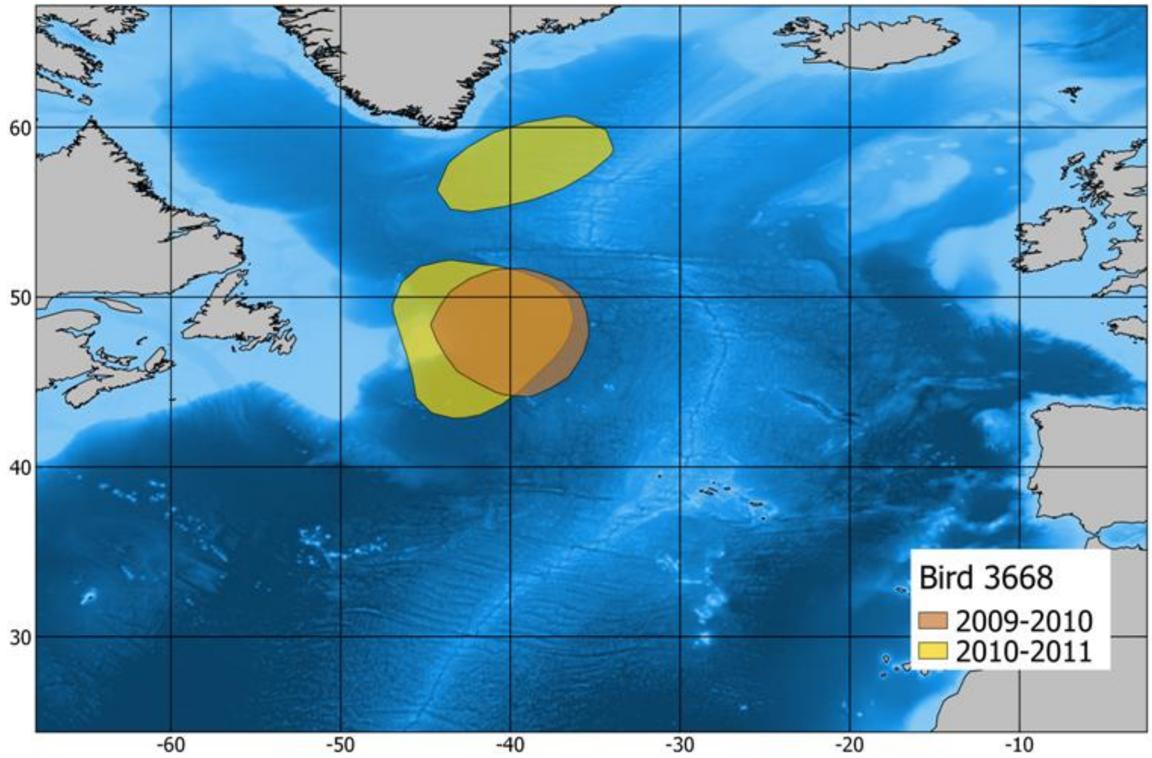

(d)

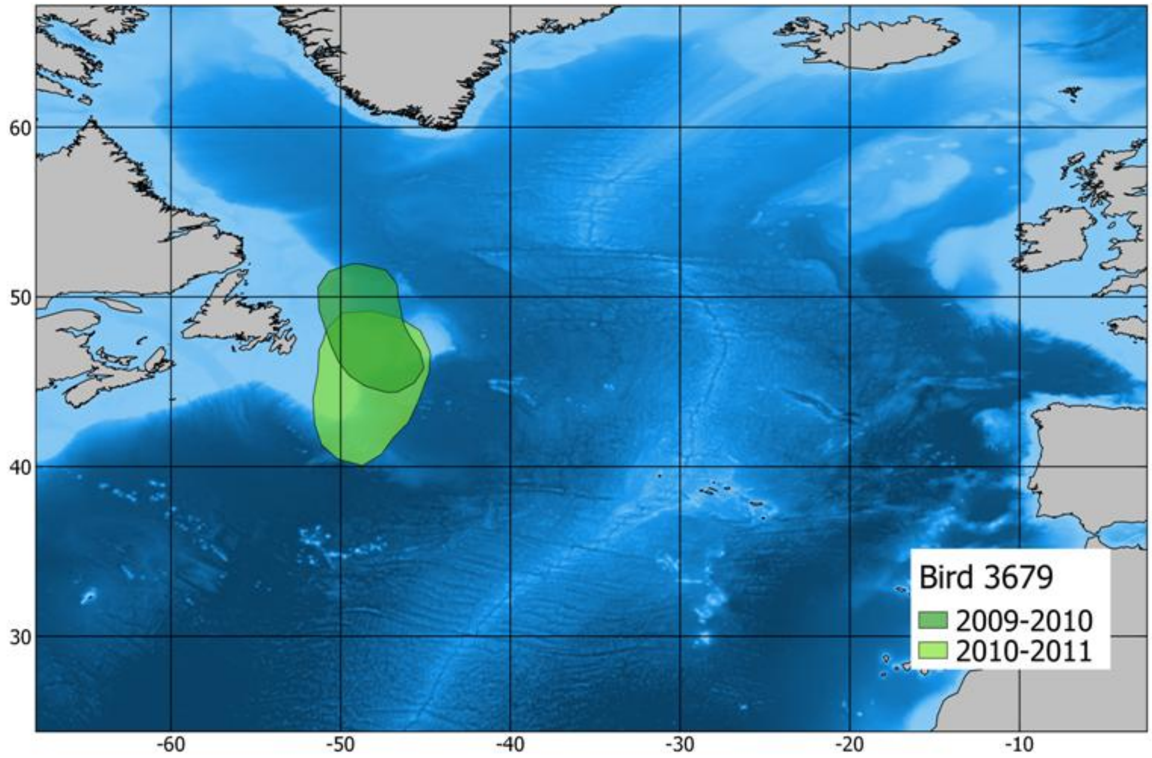

Supplement: Winter locations of four birds for which 2 (or 3) consecutive years were recorded. [file rsos171883supp4.pdf]
